# Elevated Soluble CD163 Plasma Levels Are Associated with Disease Severity in Patients with Hemorrhagic Fever with Renal Syndrome

**DOI:** 10.1371/journal.pone.0112127

**Published:** 2014-11-13

**Authors:** Junning Wang, Weijuan Guo, Hong Du, Haitao Yu, Wei Jiang, Ting Zhu, Xuefan Bai, Pingzhong Wang

**Affiliations:** 1 Department of Infectious Diseases, Tangdu Hospital, Fourth Military Medical University, Xi'an, Shaanxi Province, China; 2 Department of Obstetrics and Gynecology, Chang An Hospital, Xi'an, Shaanxi Province, China; University of Kansas Medical Center, United States of America

## Abstract

**Background:**

Hantaan virus is a major zoonotic pathogen that causesing hemorrhagic fever with renal syndrome (HFRS). Although HFRS pathogenesis has not been entirely elucidated, the importance of host-related immune responses in HFRS pathogenesis has been widely recognized. CD163, a monocyte and macrophage-specific scavenger receptor that plays a vital function in the hosts can reduce inflammation, is shed during activation as soluble CD163 (sCD163). The aim of this study was to investigate the pathological significance of sCD163 in patients with HFRS.

**Methods:**

Blood samples were collected from 81 hospitalized patients in Tangdu Hospital from October 2011 to January 2014 and from 15 healthy controls. The sCD163 plasma levels were measured using a sandwich ELISA, and the relationship between sCD163 and disease severity was analyzed. Furthermore, CD163 expression in 3 monocytes subset was analyzed by flow cytometry.

**Results:**

The results demonstrated that sCD163 plasma levels during the HFRS acute phase were significantly higher in patients than during the convalescent stage and the levels in the healthy controls (*P*<0.0001). The sCD163 plasma levels in the severe/critical group were higher than those in the mild/moderate group during the acute (*P*<0.0001). A Spearman correlation analysis indicated that the sCD163 levels were positively correlated with white blood cell, serum creatine, blood urea nitrogen levels, while they were negatively correlated with blood platelet levels in the HFRS patients. The monocyte subsets were significantly altered during the acute stage. Though the CD163 expression levels within the monocyte subsets were increased during the acute stage, the highest CD163 expression level was observed in the CD14++CD16+ monocytes when compared with the other monocyte subsets.

**Conclusion:**

sCD163 may be correlated with disease severity and the disease progression in HFRS patients; however, the underlying mechanisms should be explored further.

## Introduction

Hantaviruses are negative-sense, single-stranded, ribonucleic acid (RNA) viruses that belong to the Bunyaviridae family [1]. These viruses cause two obvious syndromes in humans: hemorrhagic fever with renal syndrome (HFRS) in Europe and Asian and hantavirus pulmonary syndrome (HPS) in the Americas. Currently, there are as many as 150,000 cases of HFRS reported annually worldwide [2]. HFRS is endemic in all 33 provinces of the People's Republic of China, where it is a significant public health problem, in which 20,000–50,000 human cases are diagnosed annually [3]. Notably, Xi'an city, which is the central district of the Shaanxi province, had an increasing HFRS incidence and mortality rate due to HFRS over the last three years [4]. There are five successive clinical phases in typical HFRS patients. These include the fever, hypotensive shock, oliguric, polyuric and convalescent phases. Additionally, some of these phases frequently overlap in severe cases, and several phases are frequently ignored in some mild cases of the disease [5]. The most remarkably pathological characteristics of HFRS are endothelial inflammation, loss of endothelial barrier function, immune cell migration and increased vascular permeability; however, HFRS pathogenesis is not entirely clear. Previous studies revealed that a “cytokine storm”, increased immune responses, complement activation and platelet dysfunction might be involved in pathogenesis of HFRS [2], [6]. Monocytes and macrophages bridging innate and adaptive immunity, high expression of cytokines activating monocytes and macrophages in the early phase of HFRS supports the immune-mediated pathogenesis [1].

Monocytes and macrophages constitute a significant component of the immune responses against viruses. These cells trigger inflammation as well as bridging innate and adaptive immunity following viral infections [7], [8]. Monocytes in circulation represent a heterogeneous population. Three major subsets of monocytes have been identified based upon the relative expression of the lipopolysaccharide co-receptor, CD14 and the FcγRIII receptor, CD16. “Classical” monocytes (CD14++CD16−, Mon1) account for 80–90% of the monocytes in circulation, whereas the “intermediate” (CD14++CD16+, Mon2) and “non-classical” (CD14+CD16++, Mon3) monocyte subsets, of which the latter two subsets are summarized as CD16+ monocytes, account for 10 to 20% of the circulating monocytes [9], [10]. The relative monocyte distribution that is observed in circulation is dynamic and changes as a function of the inflammatory and metabolic drivers that impact the differentiation between the subsets and their functions. CD16+ monocytes increase significantly in some inflammatory and immune disorders as well as in infectious conditions. These range from arthritis [11], inflammatory bowel disease [12], acute ischaemic heart failure [13], sepsis [14] to virus infections, such as HIV infection [15] and dengue fever [16].

CD163 is a specific scavenger receptor for hemoglobin/heme in vivo. It includes nine scavenger-receptor cysteine-rich domains that are located on the extracellular side of the monocytes and macrophages membrane [17]. One of its primary and well-described functions is the clearance of extracellular hemoglobin by a means of hemoglobin–haptoglobin complex endocytosis, which thus avoids the oxidative stress associated with free hemoglobin by liberating free iron, bilirubin, and carbon monoxide [18]. CD163 expression levels on monocytes down-regulation by inflammatory cytokines, such as tumor necrosis factor α (TNF-α) and granulocyte–macro-phage colony-stimulating factor (GM-CSF) [19], [20]. A soluble form of CD163 is constituted by proteolytic cleavage of the extracellular part of the protein and is shed into circulation. Increased sCD163 levels are associated with both monocyte activation and proliferation during the course of infection or inflammation [21]. The shedding of CD163 is a constitutive process, but it can be increased by various stimuli, such as lipopolysaccharide (LPS), IL-6, and IL-10 [19]. Increased sCD163 serum concentrations have been reported in patients who suffer from various infectious and inflammatory diseases, such as sepsis, tuberculosis, diabetes, acquired immune deficiency syndrome, liver disease, dengue fever, rheumatoid arthritis and hemophagocytosis [22], [23]; however, the physiological role of sCD163 was unknown, until now.

This aims of this study were to observe the CD163 expression levels on peripheral blood monocyte subsets and the plasma sCD163 levels in HFRS patients and to further analyze the correlation among CD163, sCD163 and disease severity.

## Methods

### Ethics Statement

The study protocol conformed with the Declaration of Helsinki (2008 version) and was approved by the Ethics Committee of Tangdu Hospital, the Fourth Military Medical University. Written informed consent was obtained from all study participants.

### Patients

Eighty-one hospitalized HFRS patients were enrolled in the study from October 2011 to January 2014 at Tangdu Hospital, the Fourth Military Medical University (Xi'an, China) (see Table 1). The clinical HFRS diagnosis was confirmed serologically by detection of IgM and IgG antibodies that were specific to the HTNV nucleocapsid protein [24]. Fifteen healthy volunteers were also included in the study as normal controls. Blood samples were collected from the patients during their hospitalization. All of the blood samples were separated into plasma and peripheral blood mononuclear cells (PBMC), then they were frozen at −80°C or liquid nitrogen until further use. The clinical parameters were collected during the patient hospitalizations, using routine hospital laboratory techniques.

**Table 1:** The HFRS Patient Clinical Characteristics.

	Mild	Moderate	Severe	Critical
Demographic characteristics				
Patient number	12	21	25	23
Sample number	19	39	49	38
Age (years)	40 (34–45)	41 (31–51)	44 (36–52)	39 (38–58)
Males (%)	63.6	70.8	70.0	82.6
The clinical parameters at acute stage				
sCD163 (mg/l)	2.07 (1.76–2.73)	3.31 (2.67–3.70)	3.93 (2.96–5.82)	5.03 (4.43–9.34)
WBC (×10^3^/µL)	8.2 (5.5–13.3)	10.3 (8.8–18.7)	16.8 (10.9–22.4)	16.5 (12.8–35.2)
IFN-γ (pg/ml)	40.4 (24.7–54.8)	31.7 (22.2–77.3)	63.1 (41.8–118.1)	115.4 (32.6–180.1)
IL-6 (pg/ml)	24.5 (13.6–36.4)	30.5 (19.1–59.3)	48.8 (25.4–110.0)	114.5 (38.7–216.2)
PLT (×10^3^/µL)	88.5 (62.7–103.5)	48.5 (34.3–98.3)	35.0 (23.5–44.5)	26.2 (16.3–60.2)
BUN (µmol/L)	5.5 (3.6–10.6)	15.1 (9.3–20.2)	22.7 (17.1–27.1)	16.6 (11.5–27.1)
Cr (µmol/L)	78 (69–219)	216 (135.5–299)	330 (243–544)	271 (189–398)
Monocyte (×10^9^/L)	0.68 (0.48–2.12)	1.21 (0.88–1.88)	2.31 (1.51–2.96)	2.32 (1.34–3.91)

Values represent medians with their corresponding interquartile ranges. Acute stage include the febrile, hypotensive and oliguria phases.

Abbreviations: sCD163, soluble CD163; IL-6, Interleukin-6; IFN-γ, interferon–γ; WBC, white blood cells; PLT, platelet count; BUN, blood urea nitrogen; Scr, serum creatinine.

According to the laboratory parameters and symptoms, such as body temperature, hemorrhage, edema, blood pressure and urine volume, the degree of HFRS disease severity was classified into the four clinical types, as formerly described [25]. These types were classified as: (1) mild, which included mild renal failure without an obvious oliguric stage and hypotension; (2) moderate, which included evident uremia, effusion (bulbar conjunctiva), hemorrhage (skin and mucous membrane) and acute renal failure with an obvious oliguric stage; (3) severe, which included severe uremia, effusion (bulbar conjunctiva and either pleura or peritoneum), hemorrhage (skin and mucous membrane), and acute renal failure with oliguria (a urine output of 100–500 ml/day) for ≤5 days or anuria (a urine output of <100 ml/day) for ≤2 days; and (4) critical, which were cases with one or more of the following symptoms compared with the severe patients: refractory shock (≥2 days), visceral hemorrhage, heart failure, pulmonary edema, brain edema, severe secondary infection and severe acute renal failure with oliguria (a urine output of 50–500 ml/day) for >5 days or anuria (a urine output of <100 ml/day) for >2 days, or a blood urea nitrogen level of >42.84 mmol/L. The exclusion criteria were: (1) any other kidney disease, (2) diabetes mellitus, (3) autoimmune disease, (4) hematological disease, (5) cardiovascular disease, (6) viral hepatitis (types A, B, C, D or E), and (7) any other liver disease. In addition, none received corticosteroids or other immunomodulatory drugs during the study period.

### PBMC Isolation

PBMC from the healthy controls and HFRS patients were obtained from 20 ml of heparinized venous blood using density gradient centrifugation with lymphoprep 1.077 g/ml (d = 1.077 g/ml; Sigma-Aldrich, America). The PBMCs were harvested from the interface and washed twice in RPMI-1640. The PBMC viability was >95% after Trypan Blue exclusion. The PBMCs were then aliquoted and resuspended in 1 ml fetal calf serum and freeze media (10% DMSO in RPMI-1640) and stored initially at −70°C for 24 hours before introduction into liquid nitrogen cryopreservation for later analysis. All PBMC samples were obtained at the same time as the plasma samples that were used to measure sCD163.

### Flow cytometry

Frozen PBMCs were quickly thawed in a 37°C water bath, until only a small crystal remained, and washed twice in 10 ml of heated RPMI with 10% fetal bovine serum. The thawed cells that were used for staining were incubated with 10% FCS at 37°C for 30 minutes prior to staining. The cells were then resuspended at a final concentration of 1×10^6^ PBMCs/ml. A separate aliquot of cells were stained with a cocktail composed of 20 µl CD14 (anti-CD14-FITC, clone M5E2, BD Biosciences, San Jose, CA, USA), 5 µl CD16 (anti-CD16-APC, clone B73.1 BD Biosciences, San Jose, CA, USA), and 20 µl CD163 (anti-CD163-PE, clone GHI/61, BD Biosciences, San Jose, CA, USA). The cells were incubated in the dark for 30 min at 4°C. Following incubation, the cells were washed in 2 ml of cold PBS, pH 7.4. The flow cytometric analysis was performed within 1 hour using a FACS Aria II analyzer (BD Biosciences, San Jose, CA, USA). Monocytes were identified based on their forward and side scatter appearance. We recorded 100,000 events for each sample, CD14 and CD16 were used as monocyte markers, isotype-matched controls were used according to the manufacturer's recommendations, and the percentage of positive cells for each individual marker were obtained after monocyte gating. The percentage of CD163 positive monocytes out of the total monocyte population was recorded and analyzed. The data were analyzed with FlowJo software, version 7.6.1 (Oregon, USA).

### ELISA assays

One hundred and forty-five plasma samples were obtained from 81 HFRS patients and 15 healthy control subjects and were stored at −80°C until later use. The sCD163, IL-6 and IFN-γ concentration measurements were obtained by a double antibody sandwich ELISA (Human sCD163 ELISA Ready-SET Go! eBioscience Products, Catalog Number: 88-50360; IL-6 and IFN-γ, Mabtech Products, Catalog Number: 3424-1H-2, 3460-1H-2). In this study, duplicate wells were used to detect the plasma sCD163, IL-6 and IFN-γ concentrations. Each examination step was carried out strictly following the product manual.

## Statistical analyses

The analyses were performed with the SPSS 17.0 (SPSS Inc, Chicago, IL, USA) and GraphPad Prism 5 software packages (GraphPad Software, San Diego CA). Medians and interquartile ranges were calculated for the continuous variables and numbers and percentages were calculated for the categorical variables. A Kruskal-Wallis test performed for analysis of variation among multiple groups. Mann–Whitney U tests were used to compare the sCD163 levels between the two independent groups. Correlations were compared with a Spearman's correlation analysis. All of the tests were two-sided and *P* values that were less than 0.05 were considered to be statistically significant.

## Results

### Clinical parameters and demographic conditions of HFRS patients

A total of 81 patients were confirmed to be HFRS following HTNV IgM and IgG specific antibody detection evaluations from the patients' serum specimens. From these patients, 145 plasma samples were collected during the febrile/hypotensive (Febr/Hypo), oliguric (Olig), diuretic (Diur), and convalescent (Conv) disease stages. On the basis of the clinical records and diagnostic criteria, 12, 21, 25, and 23 patients were classified as having mild, moderate, severe, and critical HFRS types, respectively. The clinical parameter specifics that were detected during the HFRS patient hospitalizations are summed in Table 1.

### Changes in the soluble CD163 plasma level in the HFRS patients

**Figure 1:**
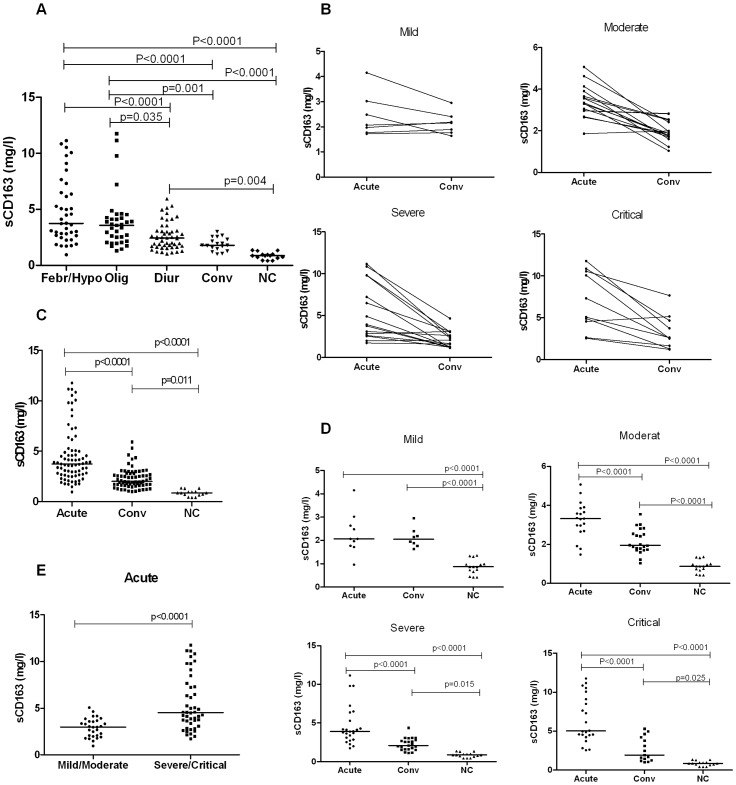
The obvious changes in the soluble CD163 (sCD163) plasma levels in the different HFRS severity patient groups. (A) Comparison of plasma sCD163 levels at the different HFRS stages in the patients. Data were obtained from 145 plasma samples that were collected during the febrile/hypotensive (Febr/Hypo), oliguric (Olig), diuretic (Diur), and convalescent (Conv) HFRS phases in the patients and from 15 healthy subject plasma samples that were used as normal controls (NC). Significant differences were observed between the following stage comparisons: febrile/hypotensive or oliguric vs. NC (*P*<0.0001); febrile/hypotensive vs. diuretic or convalescent (*P*<0.0001); oliguric vs. diuretic (*P* = 0.035), oliguric vs. convalescent (*P* = 0.001); and diuretic vs. NC (*P* = 0.004). (B) The plasma sCD163 level changes in the acute (which included the febrile, hypotensive, and oliguric stages) and convalescent phase samples (which included the diuretic and convalescent stages) of the same patient in the different disease severity groups are represented. (C) The changes in the patient sCD163 plasma levels of the acute and convalescent phases when compared with the normal controls. (D) The changes in the sCD163 plasma levels during the acute phase and convalescent phases when compare with the normal controls in the different disease severity groups. (E) Comparison of the acute phase sCD163 levels between the mild/moderate and the severe/critical groups. The significance of the differences among the multiple groups was determined with the Kruskal-Wallis test. The significance of the differences between the two groups was determined with the Mann–Whitney U test. Black lines represent medians and the *P* values are plotted in each graph.

The median sCD163 levels during the febrile/hypotensive, oliguric, diuretic, and convalescent stages, as well as in the normal controls, were 3.75 mg/l, 3.58 mg/l, 2.43 mg/l, 1.80 mg/l, and 0.81 mg/l, respectively. On the basis of the clinical disease course classification criteria, the acute phase comprised the febrile, hypotensive and oliguric stages, and the convalescent phase comprised the diuretic and convalescent stages [26]. During the acute phase, the sCD163 in the HFRS patient plasma samples was obviously higher than the levels observed in the normal controls (febrile/hypotensive or oliguric vs. NC, *P* <0.0001). The plasma sCD163 level in the HFRS patients decreased during the convalescent phase (febrile/hypotensive vs. diuretic or convalescent, *P* <0.0001; oliguric vs. convalescent, *P* = 0.001); however, it was still higher than the levels observed in the normal controls (diuretic vs. NC, *P* = 0.004) (Figure 1A). The plasma sCD163 levels in the patients with different disease severities displayed a similar change trend; however, a more distinct decline was observed in the severe/critical patient group (Figure 1B and 1D, *P*<0.0001). The sCD163 concentration in the acute phase was higher than the level that was observed during the convalescent phase in the HFRS patients (Figure 1C, *P*<0.001). The sCD163 concentration was also obviously higher during the acute and convalescent phases in the HFRS patients compared with those in the normal controls (*P*<0.0001), (Figure 1C and 1D). The sCD163 plasma levels in the severe/critical group were higher than those in the mild/moderate group during the acute (*P*<0.0001) (Figure 1E). The plasma sCD163 levels in the mild/moderate group were compared with those in the severe/critical group, and only 4 (13.7%) of the 29 mild/moderate group cases had plasma sCD163 levels that were over 4 mg/l, while 28 (60.8%) of the 46 severe/critical group cases had sCD163 levels that were over 4 mg/l (a 4.3-fold change between the high vs. mild/moderate groups). These results demonstrate that there was some kind of an association between the plasma sCD163 concentrations and the disease severity during the HFRS course.

### The correlation between the sCD163 levels and clinical parameters

**Figure 2:**
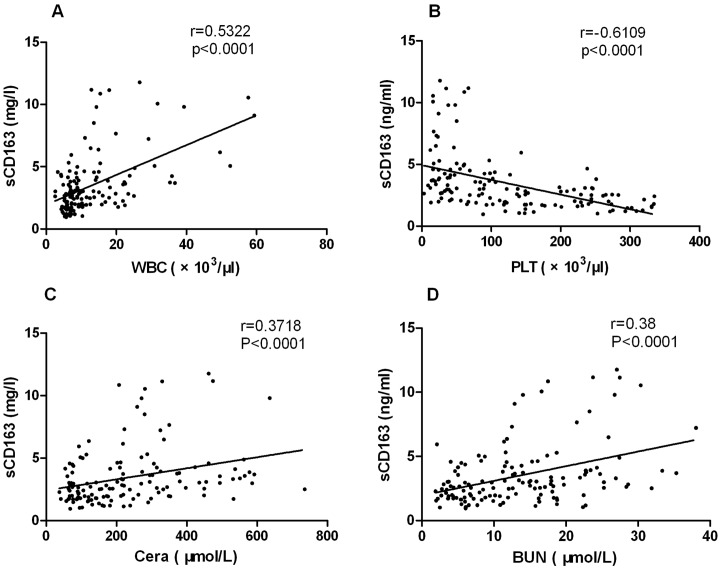
The relationship between elevated sCD163 plasma levels and the clinical parameters. The values are derived from the same blood sample from the HFRS subjects during hospitalization. The Spearman correlation test was used to test the correlation between the sCD163 levels and clinical parameters. The serum sCD163 concentrations were positively correlated with the white blood cells (WBC) (A), creatinine (Crea) (C), and the blood urea nitrogen (BUN) (D) levels for the entire group of subjects while they were negatively correlated with the platelet count (PLT) (B) levels. The r and *P* values are indicated in the graphs.

The relationships between the plasma sCD163 concentrations in the HFRS subjects and the key clinical parameters that can represent the disease severity were analyzed. A Spearman correlation analysis showed that the increased sCD163 concentration was positively correlated with the increased WBC (Figure 2A, r = 0.5322, *P*<0.0001), Crea (Figure 2C, r = 0.3718, *P*<0.0001), BUN (Figure 2D, r = 0.38, *P*<0.0001) levels, and negatively correlated with the decreased PLT counts (Figure 2B, r = −0.6109, *P*<0.0001) in the HFRS patients.

### Monocyte subsets were altered in patients with HFRS

**Figure 3:**
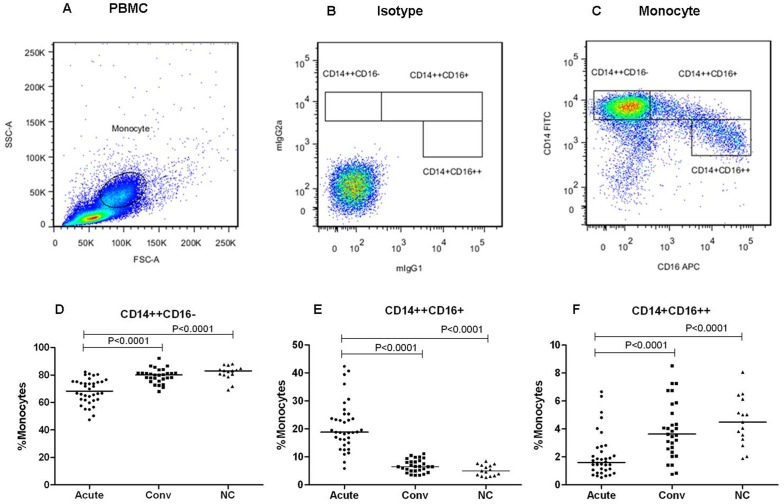
The monocyte subset proportions were altered in the HFRS subjects. A representative example of the monocyte subset analysis procedure is displayed (A, B, and C). (A) Monocytes from the total PBMC population were identified by forward (FSC) and side scatter (SSC) property analysis. (B) The isotypic negative control staining of CD14 and CD16 is displayed. (C) Using surface CD14 and CD16 expression, monocyte gated populations were further divided into three monocyte subsets, which were classic (CD14++CD16−), intermediate (CD14++CD16+) and non-classical monocytes (CD14+CD16++). A summary of the monocyte subset distribution analyses during the acute and convalescent HFRS phases and in the healthy subjects is displayed (D, E, and F). The significance of the differences among the multiple groups was determined by the Kruskal-Wallis test, Black lines represent medians and *P* values are plotted in each graph.

Monocytes and their subsets were identified by flow cytometry based on their forward and side scatter characteristics and by their CD14 and CD16 expression levels [27]. The gating strategy used to identify the classical (CD14++CD16−), intermediate (CD14++CD16+), and non-classical monocytes (CD14+CD16++) is shown in Figure 3C. Additionally, summary figures are displayed in Figures 3D, 3E, and 3F. The intermediate monocyte proportions (CD14++CD16+) were significantly increased during the patients of acute phase (median = 18.5%, interquartile range IQR = 15.3%–24.9%, *P*<0.0001) compared with the patients of convalescent phase (median = 6.5%, IQR = 5.1%–8.5%) and the healthy control (median = 5.0%, IQR = 3.3%–7.1%). However, no significant differences (*P*> 0.05) were observed between the convalescent phase and healthy control proportions, (Figures 3E). The classical (CD14++CD16−) and non-classical (CD14+CD16++) monocyte proportions were both significantly decreased during the acute phase (median = 68.4%, IQR = 61.8%–75.1%, and median = 1.6%, IQR = 1.1%–2.58%, *P*<0.0001) compared with those during the convalescent phase (median = 80.0%, IQR = 76.5%–81.6% and median = 3.6%, IQR = 2.5%–5.5%) and in the healthy controls (median = 83.0%, IQR = 80.1%–84.7% and median = 4.5%, IQR = 3.2%–6.1%); however no significant differences between both monocyte subsets (*P*>0.05) were observed between the patients of convalescent phase and the healthy controls, (Figures 3D and 3F).

### The CD163 expression on the monocyte subsets

**Figure 4:**
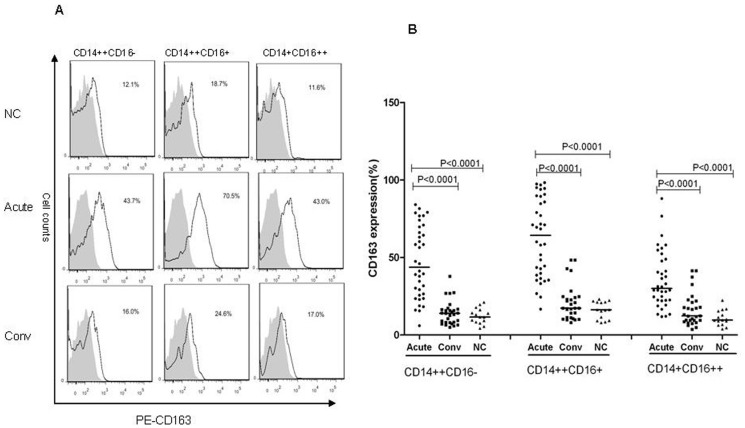
CD163 was differentially expressed on the monocyte subsets. (A) CD163 protein expression (black lines) in each monocyte subset is shown compared with the matched isotype control (shaded histograms) in the histograms. The data illustrate results from a representative experiment. (B) The CD163 expression summary data for the three monocyte subsets during the acute and convalescent phases and in the normal controls. The significance of the differences among the multiple groups was determined with the Kruskal-Wallis test. The black lines represent medians and *P* values are plotted for each graph.

As is known, the CD163 expression on the monocyte subsets surface is different [15]. In this study, we confirmed that the CD163 expression on the CD14++CD16+ monocyte subset was most highly expressed compared with less expression on the CD14++CD16− monocyte subset and lower levels on the CD14+CD16++ monocyte subset (Figure 4B). CD163 expression on the CD14++CD16+ monocyte was more remarkable during the acute phase (median = 64.2%, IQR = 38.8%–88.5%, *P*<0.0001) than during the convalescent phase (median = 17.3%, IQR = 11.3%–24.5%) in the HFRS patients and healthy controls (median = 16.3%, IQR = 9.4%–21.3%); however no significant (*P*>0.05) differences were observed during the convalescent phase and in the healthy controls. CD163 expression on the classical and non-classical monocytes were increased during the acute phase (median = 43.6%, IQR = 25.9%–68.8% and median = 30.0%, IQR = 22.4%–48.6%, *P*<0.0001) when compared with the convalescent phase (median = 14.0%, IQR = 8.1%–16.5% and median = 12.3%, IQR = 9.1%–21.1%) and healthy control findings (median = 11.6%, IQR = 8.3%–16.2% and median = 9.6%, IQR = 6.7%–15.4%); however, no significant differences (*P*>0.05) were observed in the convalescent phase and healthy control samples, (Figure 4B).

### The sCD163 levels correlated with frequency of monocytes

**Figure 5:**
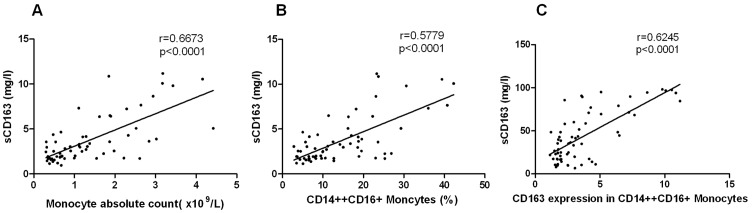
The sCD163 levels correlated with frequency of monocytes. Relationship between elevated plasma sCD163 levels and the absolute monocyte counts (A), intermediate (CD14++CD16+) monocyte percentage (B), and CD163 expression on the intermediate (CD14++CD16+) monocytes (C) evaluated by the Spearman correlation test, The values were derived from the same HFRS patient blood samples during their hospitalizations. The r and *P* values are indicated in the graphs.

There was a correlation between the sCD163 levels and the absolute monocyte counts, increased CD14++CD16+ monocyte percentage, and the CD163 expression on CD14++CD16+ monocyte. A Spearman correlation analysis demonstrated that the increasing sCD163 level was positively correlated with the increasing monocyte absolute counts (Figure 5A, r = 0.6673, *P*<0.0001), CD14++CD16+ monocytes (Figure 5B, r = 0.5779, *P*<0.0001) and the CD163 expression on CD14++CD16+ monocyte (Figure 5C, r = 0.6245, *P*<0.0001) in the HFRS patients.

### The correlation between the sCD163 levels and cytokine

**Figure 6:**
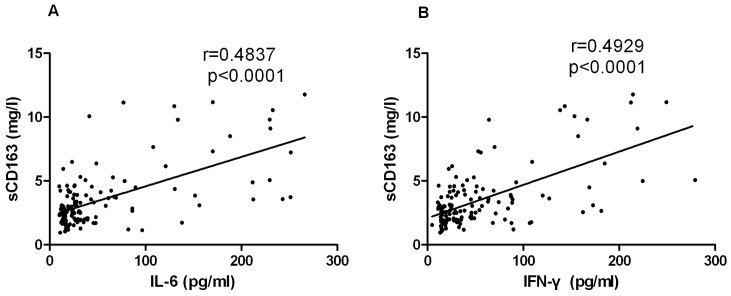
The sCD163 levels correlated with IL-6 and IFN-γ. Relationship between elevated plasma sCD163 levels and IL-6 (A) and IFN-γ (B) plasma levels evaluated by the Spearman correlation test. The values were derived from the same HFRS patient blood samples during their hospitalizations. The r and *P* values are indicated in the graphs.

There was a correlation between the sCD163 plasma levels and IL-6 and IFN-γ plasma levels in the HFRS patients. A Spearman correlation analysis showed that the increased sCD163 concentration was positively correlated with the increased IL-6 (Figure 6A, r = 0.4837, *P*<0.0001), IFN-γ (Figure 6B, r = 0.4929, *P*<0.0001) concentration in the HFRS patients.

## Discussion

Our study reports that the plasma levels of sCD163, a surface marker that is shed by monocytes, were significantly higher in HFRS patients during the acute phase than in the healthy controls and during patients of the convalescent stage. Importantly, the sCD163 plasma levels in the severe/critical group were higher than those in the mild/moderate group during the acute. The plasma sCD163 level elevation was also closely related to the clinical parameters in the present study. Additionally, we demonstrated that CD14++CD16+ monocyte ratio increased significantly during the acute phase. However, the CD14++CD16− and CD14+CD16++ monocyte ratios were reduced. Though the CD163 expression on the three monocyte subsets was increased during the acute stage, the CD163 expression on the CD14++CD16+ monocytes was the highest levels compared with the CD163 expression on the other monocyte subsets. This is consistent with research that evaluated HIV infection [15]. Additionally, the plasma sCD163 levels correlated with expansion of the CD14++CD16+ monocytes and increased CD163 expression on CD14++CD16+ monocyte. Overall, these results suggest that sCD163 is a novel marker for HFRS, and likely, the monocyte-mediated disease progression that is associated with Hantaviruses infection.

In our study, sCD163 levels were elevated during the acute stage; however, they fell gradually during the recovery stage. This is in agreement with a previous observation in malaria patients [28]. The sCD163 plasma levels in the severe/critical group were higher than those in the mild/moderate group during the acute, and interestingly, the mild/moderate group still had elevated sCD163, which suggested that monocyte activation occurs even with moderate disease during the acute stage, albeit to a lower extent. The sCD163, but not the membrane-bound form, might have anti-inflammatory functions because it inhibits T lymphocyte activation and proliferation in vitro [29]. The binding of Hb-Hp complexes to sCD163 has been shown to inhibit the supply of heme iron that is available for hemolysis that is caused by a bacterial infection [30]. Furthermore, during the innate immune response, CD163 plays a vital function in the hosts ability to reduce inflammation [31]. Interestingly, several inflammatory processes are associated with elevated sCD163 levels [31], [32], [33], [34], [35]. Because HFRS is associated with a higher inflammatory response and many inflammatory cytokines and chemokines are involved in the process, our research team [36] evaluated the serum TNF-α, IL-6, IL-4, IL-8,interferon (IFN)-γ, IP-10, and chemokine (C–C motif) ligand 5 levels in HFRS patients. The data showed that significant changes in serum TNF-α, IL-8, IFN-γ, IL-6, IP-10 and RANTES concentrations occurred in patients as they progressed through the different HFRS phases. In this experimental study, our found that increased sCD163 concentration was positively correlated with the increased IL-6 and IFN-γ. It seems that Hantaan virus infections induced over production of many inflammatory cytokines, known as a “cytokine storm” during the early stage. The increased sCD163 concentration during the acute stage most likely serves as a counter regulatory mechanism against inflammation. These results could be interpreted together with previous reports to suggest that CD163 might have a key position in the infectious diseases [23]. The membrane-bound CD163 receptor itself has anti-inflammatory capabilities by scavenging plasma hemoglobin and the products formed from toxic heme decomposition [37].

There was also a positive correlation observed between elevated plasma sCD163 levels and BUN and Cr levels, which was previously shown to predict kidney dysfunction in HFRS patients [25]. This suggests that sCD163 might be involved in renal dysfunction. In fact, studies have shown that in PUUV infected patients, acute hantavirus infection was associated with immune reaction-induced renal tissue damage [38]. Sustained inflammation can lead to renal dysfunction, and vice-versa, renal dysfunction can fuel the inflammatory response; therefore, it is not surprising that sCD163 in our study kept rising in patients who developed kidney dysfunction. Elevated sCD163 levels have been reported in chronic kidney disease patients [39]. Hemolysis, disseminated intravascular coagulation (DIC), and bleeding are characteristic clinical manifestations for HFRS [1], [24] and result in hemoglobin/heme release, which yields ferrous ions. At the same time, oxidation reduction reactions that catalyze hydrogen peroxide give rise to oxygen free radical release, which causes injury to the kidney and other organs [40]. Therefore, timely hemoglobin/heme clearance is required to avoid its excessive discharge into the blood and the outbreak of critical pathological responses. Hemoglobin/heme can only be cleared by monocyte/macrophage phagocytes when combined with haptoglobin and CD163 recognition [18]. sCD163 is shed from monocyte surfaces and, for this reason, we speculate that plasma sCD163 is likely to be highly expressed during HFRS and reflects kidney injury during this disease. However, there are no reports regarding this issue.

In the acute stage, CD163 expression on monocyte subset was increased. We showed that CD163 is more highly expressed on CD14++CD16+ monocytes compared with CD14++CD16− and CD14+CD16++ monocytes. Additionally, we showed a significant correlation between plasma sCD163 levels and the percentage of CD14++CD16+ monocytes and CD163 expression on CD14++CD16+ monocyte. This suggests that the sCD163 that is shed during Hantavirus infection is likely from CD14++CD16+ monocytes. These results may be support the hypothesis that CD14++CD16+ monocytes contribute to reduce inflammation reaction through anti-inflammatory mediator production that, in turn, inhibits T-cell differentiation and maturation. Other study found that CD14++CD16+ monocyte expansion also corresponded with disease progression in other diseases, including HIV infection [15], and in acute coronary syndrome [27]. Additionally, in the patients of convalescent phase, plasma sCD163 levels were still higher than the normal control levels; however, the membrane-bound CD163 levels were not significantly different between the HFRS and normal control groups. Thus, it is possible that plasma sCD163 also comes from activated tissue macrophages [33]. Monocytes stay in circulation for only 1 to 3 days and then migrate to peripheral tissues either spontaneously or upon stimulation [41]. Monocytes are known to migrate to the vascular endothelium, secrete cytokines, and be involved in endothelial dysfunction, which leads to increased endothelial permeability [42]. There are several conflicting reports regarding CD163 protein expression on monocytes. Some studies have shown that during chronic inflammation, surface CD163 expression is decreased [32], [43]. However, during acute inflammation, surface CD163 expression is increased [44], [45]. Additionally, through CD163 shedding, decreased surface CD163 expression was followed by the recovery and induction of surface CD163 to higher levels [46]. We speculate that surface CD163 expression might vary according to different inflammatory states in patients and during different inflammation stages.

Although we obtained some significant results, the present study has limitations. It has to be emphasized that this study only shows associations without providing evidence for possible causal mechanisms. Additionally, there were a relatively small number of patients in this study. More research is needed, with a large sample and multi-center study, to improve our insight into the physiology and time course of sCD163 release and its relation with HFRS. This may be helpful for us to further understand the pathogenesis of the disease and to offer some useful information for HFRS prevention and treatment.

In conclusion, we have identified the presence of the protein marker, sCD163, in HFRS patients, which is shed from monocytes and macrophages and is related to monocyte expansion. Additionally, sCD163 levels may be useful in predicting Hantavirus disease progression. This is the first observation that evaluated Hantaviruses infected patients with a plasma marker that is both exclusive to monocytes and an innate immune system activation marker that parallels severity and reflects the clinical parameters. Our findings indicate that sCD163 may serve as a useful biomarker for HFRS, but the underlying mechanisms should be explored further.
